# Towards Non-Invasive Methods to Assess Population Structure and Biomass in Vulnerable Sea Pen Fields †

**DOI:** 10.3390/s19102255

**Published:** 2019-05-15

**Authors:** Giovanni Chimienti, Attilio Di Nisio, Anna M. L. Lanzolla, Gregorio Andria, Angelo Tursi, Francesco Mastrototaro

**Affiliations:** 1 Department of Biology and CoNISMa LRU, University of Bari Aldo Moro, 70125 Bari, Italy; angelo.tursi@uniba.it (A.T.); francesco.mastrototaro@uniba.it (F.M.); 2 Department of Electrical & Information Engineering (DEI), Polytechnic University of Bari, 70125 Bari, Italy; attilio.dinisio@poliba.it (A.D.N.); anna.lanzolla@poliba.it (A.M.L.L.); gregorio.andria@poliba.it (G.A.)

**Keywords:** corals, Pennatulacea, *Pennatula*, model, Mediterranean Sea, biometry, ROV, trawling, fishery, VME

## Abstract

Colonies of the endangered red sea pen *Pennatula rubra* (Cnidaria: Pennatulacea) sampled by trawling in the northwestern Mediterranean Sea were analyzed. Biometric parameters, such as total length, peduncle length, number of polyp leaves, fresh weight, and dry weight, were measured and related to each other by means of regression analysis. Ad hoc models for future inferencing of colonies size and biomass through visual techniques were individuated in order to allow a non-invasive study of the population structure and dynamics of *P. rubra*.

## 1. Introduction

The mesophotic and aphotic zones of the Mediterranean Sea are inhabited by a variety of benthic organisms, some of which are able to create biogenic habitats due to their three-dimensionality and their aggregative behavior. Among them, corals play a crucial role as habitat formers, being the main builders of peculiar coral frameworks, in the case of stony corals [1], or coral forests, in the case of arborescent corals [2,3]. These habitats are featured by a high sensitivity to human pressures, particularly on trawlable grounds, where their abundance and their associated community significantly decrease [4]. Among soft-bottom octocorals, pennatulaceans can form extensive populations, known as sea pen fields, providing relevant structure in flat, low-relief muddy habitats where there is little physical habitat complexity. These fields create essential biogenic habitat for suprabenthic and benthic invertebrates, as well as an important feeding and nursery area for a rich demersal fish fauna [5,6,7,8] representing Essential Fish Habitats and Vulnerable Marine Ecosystems (VMEs) [9] worthy of protection.

Sea pen fields are often difficult to be found on muddy bottoms using indirect methods because they cannot be detected using the common habitat mapping geophysical techniques. The most effective way to identify these fields, through the analysis of data coming from commercial fishing bycatch or from experimental trawl fishing surveys, on a large scale still remains trawling. After the identification of a sea pen field, the visual techniques used onboard oceanographic cruises, such as Remotely Operated Vehicles (ROVs), allow to carry out more detailed studies and/or monitoring of VMEs on a relatively small area [10]. Non-contact and non-destructive imaging techniques, based on properly developed object segmentation and detection algorithms, have been proved to be a viable alternative to contact measurement and diagnostic techniques in a large variety of sectors, ranging from industrial quality control to characterization of devices, to medical imaging and clinical applications. Hence, it is foreseeable the development of automatic vision methods for identification, counting, and measurement of the sea [11,12,13,14,15,16,17,18,19,20].

Despite the recognized ecological importance of pennatulaceans, little is known about their biology and ecology, and their vulnerability to human pressures has been assessed under a precautionary approach [21]. Destructive sampling is still needed to estimate the biomass of a sea pen field, as well as to collect information on colonies’ size, population structure, and dynamics, in order to assess the main features of the population and to establish its need for protection. Nowadays, advances in the design, lowering of costs, and increased performance of unmanned vehicles, including ROVs and drones, pave the way to the extended exploration of marine environments [22,23,24,25,26,27,28]. Underwater imagery techniques, such as ROVs and towed cameras, are now allowing a better understanding of the sea pen numerical importance on a relatively small scale, but these methods are still not effective in understanding biomass and size structure [10]. Hence, the development of a non-invasive technique for determining the weight of colonies is desirable to obtain quantitative data of biomass (both fresh and dry) from ROV imaging, useful to support conservation measures. Moreover, the possibility to estimate the colonies’ length with the same approach would also enhance the size structure assessment of sea pens population and their monitoring, consistently with their need of protection and their fragility to the trawled sampling gears (e.g., trawl nets and dredges). Finding of proper biometric correlations could avoid the need of further sampling for the study of these vulnerable populations in the near future [29], allowing non-invasive methods, and representing a valid alternative to destructive sampling.

**Figure 1:**
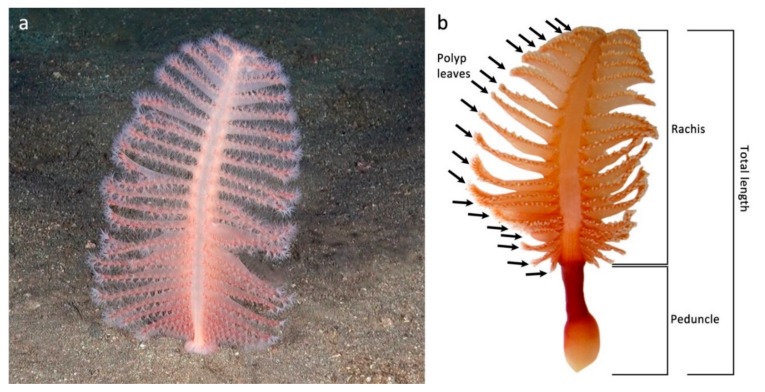
The colony of *Pennatula rubra*. (**a**) In vivo appearance of the species; (**b**) indication of polyp leaves (black arrows), rachis, peduncle, and total length.

The present study modeled the biometric measurements collected from a population of the red sea pen *Pennatula rubra* (Ellis, 1761) sampled by trawling. This species, endemic of the Mediterranean Sea, belongs to the suborder Subsessiliflorae because of the presence of polyps disposed in pinnately arranged leaves. It represents one of the most important field-forming sea pens of the Mediterranean continental shelf [30,31], reported as vulnerable in the Red List of the International Union for the Conservation of Nature (IUCN) [32] among the seventeen threatened coral species of the basin. The colonies of *P. rubra* live with the peduncle (i.e., the basal part of the colony) into the sediment, the rachis representing most of the visible portion of the colony (Figure 1). For this reason, the total length cannot be directly measured through visual methods. In this study, we found reliable biometric relationships using the number of polyp leaves as a proxy to estimate the total length and the biomass of the colonies, enabling future in situ assessments of population structure and biomass. This would also avoid the need for sampling for the study of the population dynamic of *P. rubra*, representing a necessary knowledge base for a non-invasive study of the wild populations.

## 2. Materials and Methods

Colonies of *P. rubra* were sampled using an experimental trawl net, with a stretched mesh of 20 mm in the codend, in the frame of the MEDITS (Mediterranean International Bottom Trawl Survey) project [33]. Sampling was carried out during 2013 northwest Punta Alice (Ionian Sea, southern Italy; start: 39°35.05′N–16°52.26′E; end: 39°34.05′N–16°53.63′E) at 61–65 m depth [31], onboard the *Pasquale e Cristina* fishing vessel. A SCANMAR acoustic system (Scanmar AS, Åsgårdstrand, Norwey) [34] was used to measure the horizontal and vertical openings of the net in order to estimate the swept area. The colonies of *P. rubra* sampled were preserved on board at −20°C.

A total of 168 colonies, sampled over an area of 41,000 m^2^, were analyzed. The following biometric parameters were measured for each colony: length of the peduncle, the total length of the colony (considering both rachis and peduncle), fresh weight, and number of polyp leaves (Figure 1). Length measures were carried out using a manual caliber with 1 mm resolution, and fresh weight was measured using a DENVER MXX-212 electronic balance (Denver Instrument GmbH, Goettingen, Germany; 0.01 g resolution, 0.04 g worst-case uncertainty).

Measurements were carried out in the laboratory after thawing, considering that the freezing process causes the complete contraction of the colonies. On the contrary, living colonies can contain a highly variable quantity of seawater driving their contraction and considerably changing their size and fresh weight [31]. Then, a suitable procedure to obtain dry weight measurement was performed for a reduced number of sampled colonies. In particular, 54 colonies were selected having different fresh weight values to obtain a statistically significant population. Each colony was identified by a unique ID and fresh weighted, and then the colonies were dried in an oven at 40 °C for 96 h. Dry weight was measured for each colony. A detailed study highlighting the relationship among all the biometric parameters of *P. rubra* was performed, with the aim to develop suitable models for the colonies’ size and fresh weight based on the number of polyp leaves. The model for the estimation of size was developed to obtain both the rachis length and the total length, the former being visible with imagery techniques (Figure 1), while the latter used in direct measurements from samples. Moreover, the relationship between fresh and dry weight of the colonies was also assessed. Finally, data obtained from an ROV survey carried out on the same population sampled by trawling [10] were used to compare the distribution of the number of polyp leaves obtained from both the methods (i.e., visual vs. sampling). In particular, polyp leaves were counted for a total of 207 colonies observed in vivo, whose position, contraction, and ROV framing allowed to clearly distinguish the polyp leaves of at least one side of the colony.

Although it is not very common, the number of polyp leaves from the two sides of the same colony can be different due to mechanical damage or predation events. For this reason, both right and left polyp leaves of each sampled colony were counted. When the number of leaves was different between the two sides of the same colony, the mean value was calculated and used for the models. Whereas, the maximum number of polyp leaves per colony side was preferred over the mean value for the size estimation model, considering the direct link between the length of the colony and the number of polyp leaves.

## 3. Results and Discussion

The number of polyp leaves proved to be a reliable proxy to estimate size and biomass of *P. rubra* colonies using non-invasive approaches based on ROV imaging, as previously highlighted with regression analysis [10,29]. In particular, the analysis of colonies sampled through experimental trawl fishing surveys allowed to identify suitable relationships, which were able to estimate the size and biomass of sea pens using models based on the number of polyp leaves, that can be measured from images.

### 3.1. Model for Size Estimation

**Figure 2:**
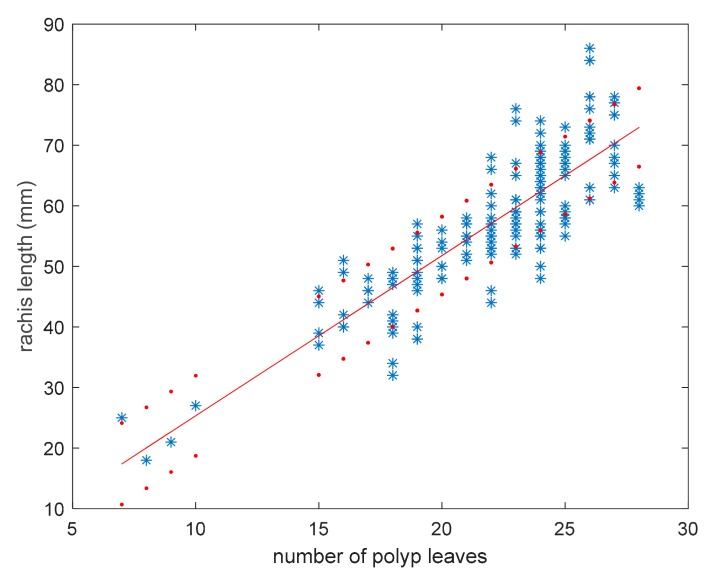
Rachis length vs. the number of polyp leaves in *Pennatula rubra*; the red line is the linear regression, and red dot lines include 95% of data.

In the first step, the relationship between rachis length and the number of polyp leaves was investigated. The experimental results highlighted a good linear behavior (correlation coefficient = 0.84) between number n of polyp leaves and the estimated rachis length as reported below in Equations (1) and (2) and shown in Figure 2.

(1) $$l_{r}=l_{t}-l_{p}$$

(2) $${\hat{l}}_{r}\left(n\right)=2.6\cdot n-1.1$$

where $l_{t}$ is the total length of the colony, $l_{p}$ is the length of the peduncle, *n* is the number of polyp leaves, and $l_{r}$ and ${\hat{l}}_{r}$ are rachis length and its estimation by means of linear regression, respectively.

To quantify the accuracy of the proposed model, the root mean square relative error $e_{l_{r}}$ was calculated by using Equation (3), then a value of 11.8% for this parameter was obtained. This value is due to intraspecific variability, and it can be accepted for size-frequency distribution analysis in soft coral populations.

(3) $$e_{l_{r}}=\sqrt{\frac{1}{M}\overset{M}{\underset{i}{\sum }}{\left(\frac{{\hat{l}}_{r}\left(n_{i}\right)-l_{r_{i}}}{l_{r_{i}}}\right)}^{2}}$$

In Equation (3), *M* is the number of samples, $n_{i}$ and $l_{r_{i}}$ are the values of polyp leaves number and rachis length of the *i*-th sample, respectively. Experimental values are equally distributed around the regression line (Figure 2).

**Figure 3:**
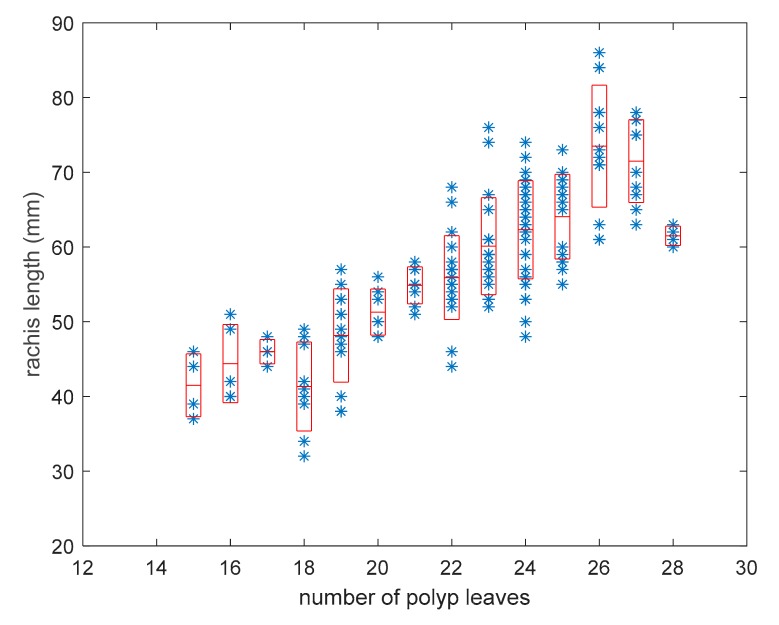
Distribution of rachis length in the population of *Pennatula rubra*; red boxes include all values ranging in average ± standard deviation.

Figure 2 shows a significant variation in colony length for each value of the number of polyp leaves; therefore the dispersion of obtained data was analyzed. Figure 3 shows the distribution of $l_{r}$ values grouped for each n, where mean and standard deviation were represented with a box plot. The values with n < 15 were not included in the dispersion analysis because they did not provide length variation in correspondence of the same values of n. In general, colonies with a high number of polyp leaves provide more dispersion, with a maximum standard deviation value of 8.2 mm. This can be due to intraspecific variability, particularly evident in older colonies whose number of polyp leaves is higher.

Similar linear dependence was obtained by analyzing the behavior of total length estimate ${\hat{l}}_{t}$ as a function of the number of polyp leaves, reported in Equation (4), with an obtained root mean square relative error of 10.6%.

(4) $${\hat{l}}_{t}\left(n\right)=5.8\cdot n+8.1$$

The major or minor variation of rachis length for each value of the number of polyp leaves can be due to the natural variability of the population and the number of colonies sampled. Despite a large number of samples analyzed (168 colonies), it is expected that the rachis length variation among the different values of the number of polyp would be more homogeneous by analyzing a larger number of colonies.

### 3.2. Model for Fresh Weight Estimation

**Figure 4:**
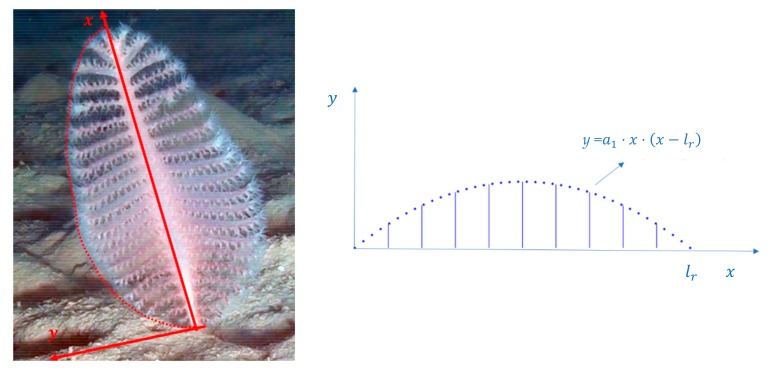
2D outline of *Pennatula* *rubra*.

The number of polyp leaves was related to the fresh weight of *P. rubra* colonies and served as a model to assess colonies’ biomass. A suitable *P. rubra* envelop was considered in order to identify the best curve for data fitting. In particular, by supposing a 2D outline of *P. rubra*, it is possible to consider a second order enveloping curve expressed as a function of distance along the rachis, as shown in Figure 4. The curve intersects the rachis at distances zero and $l_{r}$.

**Figure 5:**
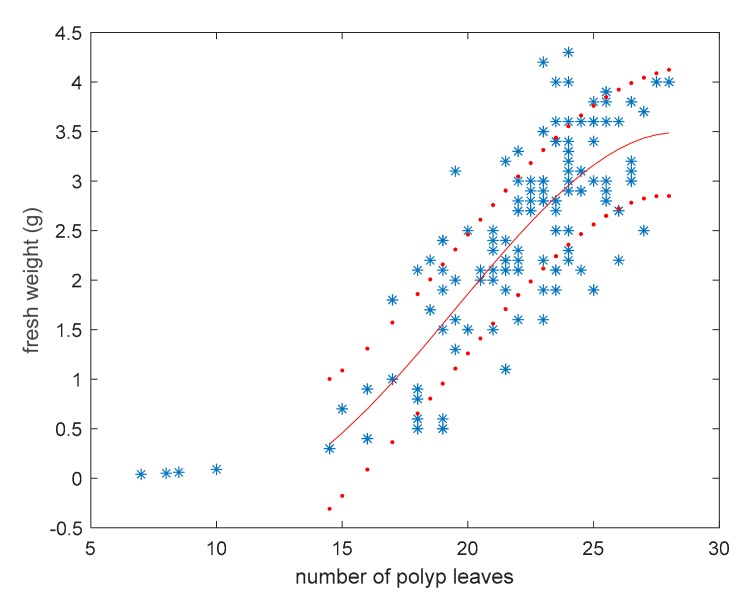
Fresh weight vs. the number of polyp leaves in *Pennatula rubra*; red curve is the third-degree polynomial fitting experimental data, and red dot curves include 95% of data.

The weight ${\hat{w}}_{f}$ of a *P. rubra* colony is assumed to be proportional to the enveloping area, and consequently can be expressed by integrating that curve between 0 and $l_{r}$, obtaining a third-degree power of $l_{r}$. Therefore, by taking into account the linear dependence between length and the number of polyp leaves, the dependence of fresh weight from n can be described by a third-degree polynomial, where all powers of n up to the third have been considered for generality (Figure 5).

Tests consisting of the use of different nonlinear models confirmed that the best fitting curve providing the minimum mean square error is expressed by the following relationship, corresponding to a root mean square relative error of 46%:

(5) $${\hat{w}}_{f}\left(n\right)=-0.013\cdot n^{3}+0.076\cdot n^{2}-1.170\cdot n+5.342$$

The validity of the model was not assured when n<15 because the weight estimated in this condition required a higher number of data from juvenile or young colonies and a more accurate weight measurement with very low values. Anyway, small colonies resulted infrequent with both trawling and visual methods, and their contribution to the biomass of the sampled population is about 0.06% of the mass.

**Figure 6:**
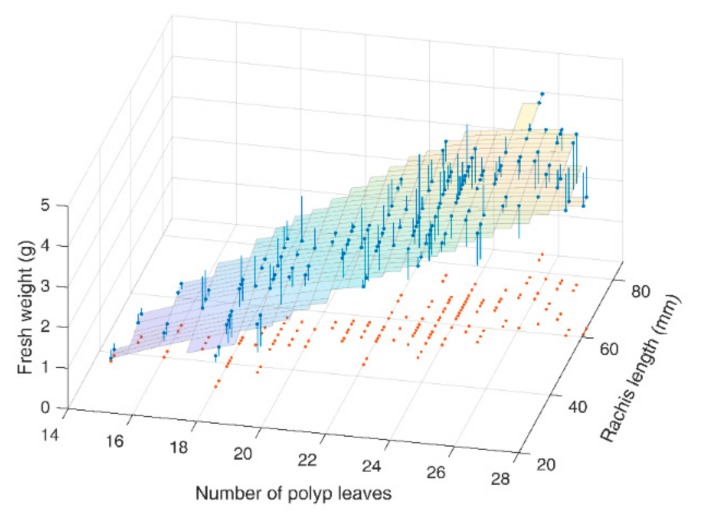
The behavior of fresh weight as a function of both the number of polyp leaves and rachis length in *Pennatula rubra*. Blue bars represent errors between measured weight and least squares surface fitting. Red dots are projections on the horizontal plane.

A further model of the estimated weight ${\hat{w}}_{2f},$ taking into account also the linear dependence on rachis length, was considered with the aim to reduce the fitting error (Equation (6)). The following fitting surface was identified, as shown in Figure 6.

(6) $${\hat{w}}_{2f}\left(n,l_{r}\right)=2.28\cdot 10^{-4}\cdot n^{3}-0.023\cdot n^{2}+0.826\cdot n+0.039\cdot l_{r}-9.182$$

In this way, the root mean square relative error was reduced to 38.7%.

### 3.3. Dry Weight Estimation

The correlation between dry and fresh weight was analyzed based on measurement data on a reduced set of *P. rubra* samples represented by 54 colonies. Despite the fact that the dry weight is not frequently used for sea pens, it is considered a more reliable measure of the biomass because of the variable water content in living colonies of *P. rubra*, driving their exposure to different currents and their withdrawal as a defense strategy [31].

**Figure 7:**
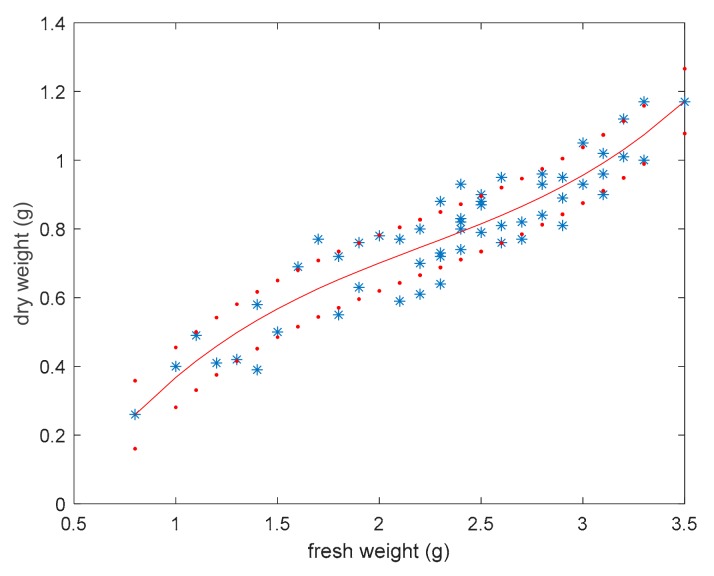
Fresh weight vs. dry weight in *Pennatula rubra*; the red curve is the polynomial interpolation of the third order, and red dot curves include 95% of data.

Figure 7 shows fresh weight behavior vs. dry weight, which can be described by means of a polynomial function of the third order expressed by means of

(7) $${\hat{w}}_{d}\left(n\right)=0.062\cdot w_{f}{}^{3}-0.412\cdot w_{f}{}^{2}+1.134\cdot w_{f}-0.416$$

where $w_{f}$ is the measured fresh weight, and ${\hat{w}}_{d}$ is the dry weight estimation.

The obtained model allows to predict dry weight based on the fresh weight and shows root mean square relative error of 19.2%. This can allow the use of the model to assess dry biomass starting from both fresh weight estimation (e.g., from ROV imaging) or direct fresh weight measures (e.g., from fishery samples). In this last case, the model would help to rapidly understand the dry biomass of a sea pen field (e.g., onboard a fishing vessel) without the need of drying procedures.

### 3.4. Distribution of the Number of Polyp Leaves

Starting from ROV imaging data, $n_{i}$, the number of polyp leaves of the i-th colony, was counted for each of the M= 207 *P. rubra* colonies observed and analyzed, giving the set $n={\left[n_{i}\right]}_{i=0,\ldots ,206}$. This set was compared with the one obtained by trawling, consisting of M = 168 colonies, $n={\left[n_{i}\right]}_{i=0,\ldots ,167}$. 

**Figure 8:**
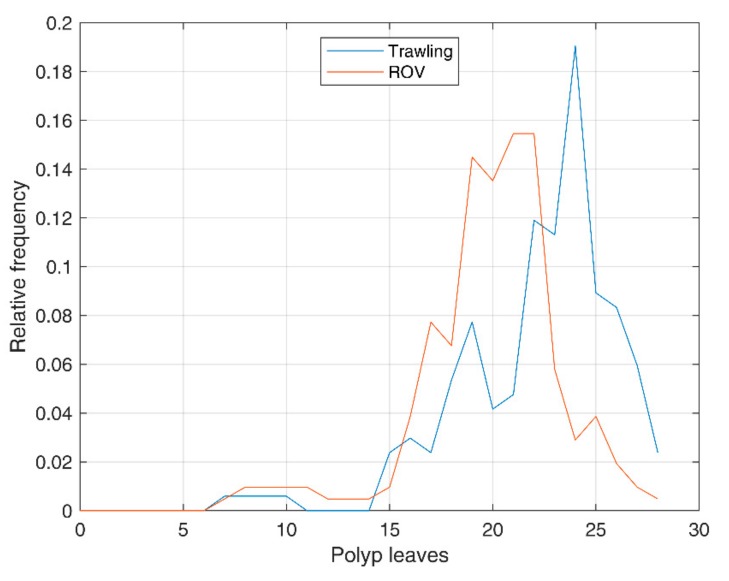
Comparison of the distribution of the number of polyp leaves in *Pennatula rubra*: in blue, samples obtained with trawling; in red, observations from Remotely Operated Vehicle (ROV) surveys.

The distributions of the number of polyp leaves in both sets are compared in Figure 8, while descriptive statistics are reported in Table 1.

Bias-corrected standard deviation, standard deviation, bias-corrected skewness, and bias-corrected kurtosis have been defined, respectively, as follows:

(8) $$\sigma =\sqrt{\overset{M-1}{\underset{i=0}{\sum }}\frac{{\left(n_{i}-\mu \right)}^{2}}{M-1}}$$

(9) $$\sigma^{\prime }=\sqrt{\overset{M-1}{\underset{i=0}{\sum }}\frac{{\left(n_{i}-\mu \right)}^{2}}{M}}$$

(10) $$s=\frac{\sqrt{M\left(M-1\right)}}{\left(M-2\right)}\overset{M-1}{\underset{i=0}{\sum }}\frac{\frac{1}{M}{\left(n_{i}-\mu \right)}^{3}}{\sigma^{\prime 3}}$$

(11) $$k=\frac{M-1}{\left(M-2\right)\left(M-3\right)}\left[\left(M+1\right)\overset{M-1}{\underset{i=0}{\sum }}\frac{\frac{1}{M}{\left(n_{i}-\mu \right)}^{4}}{\sigma^{’4}}-3\left(M-1\right)\right]+3$$

Standard deviation and kurtosis are similar for trawling and ROV data, while mean values and skewness differ by about 10% and 18%, respectively (Table 1). These differences can be due to many causes, such as: a slight difference in the two nearby subpopulations studied, since trawling hauls and ROV transects do not completely overlap; a minor catch efficiency of the trawl net on smaller colonies, that can be easily passed by the net without being collected; underestimation and errors in counting the number of polyp leaves from video analysis, considering that the first and the last polyp leaves can be very small, and their identification can be less easy from images than from samples.

By using the previously identified relationship between the number of polyp leaves and fresh weight of the colonies, an estimation of the total weight can be obtained from ROV images. In particular, for each colony i, the predicted fresh weight is ${\hat{w}}_{i}=\hat{w}\left(n_{i}\right)$ according to the model (5). ${\hat{w}}_{i}$ has been set to 0 for small colonies (n≤15), where the model is not applicable.

Based on ROV images, the predicted mean weight of a colony is

$${\hat{w}}_{mean}=\frac{1}{M}\overset{M-1}{\underset{i=0}{\sum }}{\hat{w}}_{i}=1.86\;g$$

The total weight of a population present on an area S can then be estimated through

$${\hat{w}}_{tot}={\hat{w}}_{mean}\delta S$$

where δ is the density of the colonies present on the surface S.

The mean weight of trawling samples, obtained by averaging the weights measured in the laboratory, is $w_{mean}=2.39\;g$. Therefore, the relative error of weight prediction with ROV, for a given surface density and area, is −22%. This result takes into account the previously mentioned error contributions, as well as the weight modeling error.

The weight estimation based on the number of polyp leaves through ROV imaging proved to be a feasible alternative to destructive sampling. Considering the low catch efficiency of trawl nets on sea pens [10,35,36], it cannot be excluded that small colonies have less possibility to be sampled, thus justifying the 22% difference observed between the mean weight of samples with the two methods.

## 4. Conclusions

Zoological and ecological studies on VME indicator taxa are often based on abundance, biomass, and size structure of the population studied. This information is fundamental to assess the extent and the main features of soft-bottom coral communities, such as sea pen fields and coral gardens, in order to plan and apply proper protection initiatives. Except for abundance, considered as colonies density, whose estimation is known to be more accurate using visual methods than trawled sampling gears, the gathering of both size and biomass data has been historically carried out through destructive sampling. This study showed that the number of polyp leaves could be used for non-invasive studies of vulnerable species, such as *P. rubra*. In fact, this information can be retrieved from ad hoc ROV surveys, with a good level of accuracy and good reliability.

Despite the fact that the estimation of colonies’ size and biomass using ROV imaging is a time-consuming process compared to the direct measurement of the samples, it can allow a non-destructive study and monitoring of vulnerable and protected species, for both studying and preserving the natural population. Data from different populations of *P. rubra* within the Mediterranean Sea would contribute to improve and refine the models, in order to make them applicable on a basin scale, as well as to highlight potential morphometric differences among the populations.

The use of polyp leaves to estimate other biometric parameters could be used worldwide for other species belonging to the suborder Subsessiliflorae, being characterized by the presence of polyp leaves. The identification of proper biometric relationships has been recently done for other sea pen species in eastern Canada [21] and is common for size–frequency distribution of other octocorals [37], as well as for other marine invertebrates [38]. However, this approach cannot currently be applied to rare species, such as the endemic sea pen *Crassophyllum thessalonicae* Vafidis & Koukouras, 1991 [39] and the wip-like sea pen *Protoptilum carpenteri* Kölliker, 1872 [40].

Records of benthic species collected from accidental catches (e.g., fishery) could be used to obtain useful preliminary information to identify VMEs over very large areas. The analysis of large-enough sets of samples can also represent a basis to build further ad hoc models for the future study and monitoring of these species, particularly concerning soft-bottom coral populations. This sustainable approach supports the restrictions that should take place after the finding of dense populations of vulnerable species, such as the adoption of encounter protocols and the establishment of no-fishing areas on VMEs.

## Figures and Tables

**Table 1:** Statistics of the number of polyp leaves of *Pennatula rubra* for trawling- and Remotely Operated Vehicle (ROV)-based sampling.

Set	Mean μ	Standard Deviation σ	Skewness s	Kurtosis k
Trawling	22.0	3.7	−1.3	5.6
ROV	19.9	3.5	−1.1	5.5
